# Physical–chemical analysis of different types of flours available in the Romanian market

**DOI:** 10.1038/s41598-023-49535-x

**Published:** 2024-01-09

**Authors:** Katalin Bodor, József Szilágyi, Brigitta Salamon, Orsolya Szakács, Zsolt Bodor

**Affiliations:** 1 Department of Bioengineering, Faculty of Economics, Socio-Human Sciences and Engineering, Sapientia Hungarian University of Transylvania, Libertății Sq. 1, 530104 Miercurea Ciuc, Romania; 2 Institute for Research and Development in Game Management and Mountain Resources Miercurea Ciuc, st. Progresului 35B, 530240 Miercurea Ciuc, Romania; 3 Faculty of Natural Sciences, Doctoral School of Chemistry, University of Pécs, Ifjúság 6, 7624 Pécs, Hungary; 4 Department of Food Engineering, Faculty of Economics, Socio-Human Sciences and Engineering, Sapientia Hungarian University of Transylvania, Libertății Sq. 1, 530104 Miercurea Ciuc, Romania

**Keywords:** Chemistry, Engineering

## Abstract

The physical–chemical characteristics of different types of flours are essential for understanding their composition, nutritional value, and functional properties as well. The aim of this research was to identify the variability of the different wheat flours available in Romania. In this study 39 different wheat flours were selected and the following parameters were analyzed in the laboratory: moisture content, ash content, gluten content (wet and dry) and wet gluten spreading. The tested flours were classified into four different classes according to their ash content: 480 (ash content 0.48%) (N = 11), 550 (0.55%) (N = 9), 650 (0.65%) (N = 8), 1100 (1.1%) (N = 11). Mathematical and statistical methods were used to analyze the obtained results: descriptive statistics, box-plot, Spearman correlation and hierarchical cluster analysis. The results revealed that moisture content varied between 9.5 and 11.8%. In terms of ash content, the lowest and highest measured values were 0.427–2.04 g/100 g. The average wet gluten content of the studied flours varied between 30 and 32%, while the average dry gluten content was 12.8%. The findings indicate that the moisture content of all examined flour samples was within permissible levels for extended storage, aligning with established standards. Gluten is a key and essential parameter for bread making because influences the dough mixing and baking properties. The mineral content, represented by ash content, is influenced by cereal type and milling process, with wheat's ash content ranging between 1.5 and 2%. Flours with high wet gluten content (> 34%) can be used to improve the properties of lower quality flours. Further studies are necessary in order to determine the possible health effects of different cereal varieties.

## Introduction

The cereals consumption for human and animal nutrition has been essential from ancient times to the present day^1^. Globally, wheat (*Triticum aestivum*) is considered the most valuable cereal crop, and thus the most important in terms of area under cultivation^2^. With the accelerated growth of the world population, living standards are also changing, and as our needs increase, the cultivation area of wheat is increasing year by year. According to the FAO (Food and Agriculture Organization of the United Nations) 2020 report, the world wheat production exceeds 700 million tons. The Central Statistical Office (CSO), reported in 2020 that the amount of wheat grown in the European Union was 126,659 tons^3^. Analyzing the situation in Hungary and Romania, statistics for 2021 show that wheat harvested in Hungary accounted for 38.7% of total cereals grown, or 5.3 million tons^4^. In Romania, the annual domestic wheat production averaged around 10 million tons in drought-free years (2017–1019).

Similar to the other cereals, it is carbohydrate-rich (starch 63.07%, sugar 4.32%, cellulose 2.76%), low-protein (16.06%), herbaceous agricultural crop. It is a monocotyledonous bread grain of the grass family with a low-fat content (2.24%). Wheat has an ash content of approximately 2.18%, and the main mineral trace elements are iron (33 ppm), copper (4.50 ppm), zinc (25.8 ppm) and the macro elements calcium (437 ppm) and magnesium (1100 ppm). Wheat also contains vitamins and enzymes (α-amylase, β-amylase, proteases)^5,6^ and the distribution of minerals is mainly in the husk and germ.

Among the proteins found in wheat, we can distinguish simple and complex proteins. Albumin, globulin, prolamin (gliadin) and glutelin (glutenin) can be classified as simple proteins^7^. The proportion of gliadin and glutenin is important for the quality of gluten, gliadin is responsible for the elasticity and stickiness of gluten, while glutenin is responsible for the strength and resistance of gluten. The ideal gliadin:glutenin ratio is 3:1^8^.

The most important complex protein is nucleoprotein, which is found mainly in wheat germ. The protein to starch ratio is an indicator of the quality of the wheat grain, the higher the protein ratio, the better the quality of the wheat^9^. The main components of the reserve proteins are found in the prolamin and glutelin fractions. The role of these compounds is to provide nitrogen and sulfur atoms and carbon skeletons for the germ plant^10,11^.

The beneficial effects of the water-soluble fiber in flour include helping to maintain and restore normal intestinal function and increasing its volume due to its fluid retention capacity, thus shortening the time that waste material remains in the intestinal tract and improving peristaltic movement of the intestine^11^.

To produce different bakery products, it is essential to use the specific flour type. Flours with a low ash content can be used for all purposes, and flours with a higher ash content are suitable for the production of diet products^12,13^. Several studies worldwide have focused on the analysis of the physico-chemical properties of flours and their optimal use in the production of target products^5,14–17^.

The aim of this research was to determine, classify and compare the physico-chemical parameters of commercially available wheat flours in Romania. On the other hand, it was also the objective of the research to use our quantitative and qualitative data to help consumers in their product choice and to identify the variability between different types of wheat flour as well as between the same types of wheat flour.

## Materials and methods

**Studied area**

The flours used in this study were purchased from various supermarkets located in Miercurea Ciuc, Romania.

## Physical and chemical determination of the wheat flour

Prior to the research, we purchased commercially available wheat flour in Romania in June 2021:

1. White 000 wheat flours: (Băneasa, Baromir, Băneasa (loaf), Pambac, Raftul Bunici, Arpis, Grania, Gobé, Lido Garbea, Belbake, Harmopan),
2. White 550 wheat flours: Gyermelyi, Bácskai, Deliza (pizza), Pambac, Családi, Kunsági, Elan BFF, Elan, Gyermelyi pizza.
3. White 650 white wheat flours: Pambac, Băneasa, Titan, K classic, Tótkomlósi, Góbé, Belbake, Harmopan.
4. Whole meal wheat flours: Băneasa, Natural, Titan, Nagyi Titka, Góbé, Belbake, Harmopan.

Laboratory analyses were carried out in the Sapientia Hungarian University of Transylvania Food Engineering Department Laboratories, using triplicate replicates for the following parameters: moisture content, ash content, gluten content (wet and dry) and wet gluten spreading. The laboratory methods used to determine the physical and chemical properties and the methods used for data processing are described below.

### Determination of the moisture content of flours

To determine the moisture content of the flours the samples were weighed to two decimal places using an analytical balance and dried in a drying oven at 103 ℃ to constant weight (> 4 h). After drying, the sealed samples were placed in a desiccator and weighed after cooling. The moisture content was calculated according to Eq. (1)^18,19^.

1 $${\text{Hum, \% }} = \frac{{{\text{W}}1 - {\text{W}}2}}{{\text{m}}} \times 100$$

where: W1 = mass of the sample and of the dish before drying (g), W2 = mass of the sample and of the dish after drying (g), m = mass of the sample weighed for drying (g).

The moisture content of different types of flour must be under 14.5%.

### Determination of ash content

The wheat flour samples were burnt in an oven at 550 ℃. The total mass of the crucible and the ash was measured to two decimal places after cooling in a desiccator. The crude ash content was calculated using Eq. (2), expressed in weight percent^18,19^.

2 $${\text{Ash}}_{{{\text{crude}}}} ,{\text{\% }} = \frac{{{\text{m}}^{1} - {\text{w}}^{2} }}{{{\text{m}}^{0} }} \times 100$$

where: m^0^ = mass of the sample weighed for the test (g), m^1^ = mass of the crucible and sample after incineration (g), m^2^ = mass of the crucible (g).

The total ash content was determined, based on the humifity content of the samples, using the following equation:

3 $${\text{Ash}},{\text{\% }} = {\text{Ash}}_{{{\text{crud}}}} {*}\left( {100 - {\text{Hum}}} \right){*}100$$

where, Ash_crude_ = crude ash content, Hum = the samples humidity.

Based on the ash content, the following types of flour are distinguished by the specification: where the ash content is represented in maximum %^19^.

1. White flour Type 480 = 000 (0.48%)
2. White flour Type 550 (0.55%)
3. White flour Type 650 (0.65%)
4. Semi-white (0.66–0.90%)
5. Black flour (0.91–1.4%)
6. Black dietary (1.41–2.2%)

### Wet gluten and spread determination

24 g of flour was kneaded into a dough with water, and the water-soluble substances were washed through a sieve with a water jet. The remaining water-insoluble matter is the percentage of wet gluten. The amount of gluten is a very important quality indicator for wheat varieties. A higher quality flour can improve a flour of lower quality. In addition to the amount of gluten, the area of gluten is also a very important quality indicator.

To determine the area of wet gluten, 5 g of wet gluten was weighed, manually shaped into a sphere and placed on a glass plate. Using millimeter paper placed under the glass plate, the initial diameter was determined, and then the final diameter of the gluten ball was also determined by keeping it in a drying oven at 38 ℃ for 1 h. The difference between the diameter of the gluten ball measured at the final time and the diameter of the gluten ball measured at the initial time is the gluten spreading^19^.

Based on the wet gluten the following classification was taken into account (Table 1):

**Table 1:** Different types of wheat classification based on the gluten content.

	Repair wheat	Milled wheat			Durum wheat	
		I	II:	III:	I	II:
Wet gluten, % (w/w)	34	30	28	26	32	30
Wet Gluten spreading, mm/h	2–5	3–8	3–8	–	2.5	2.5

The dry gluten content was calculated from the wet gluten content. The wet gluten was dried at a constant weight at 105 °C, and calculations were carried out by using the following equation, Eq. (4).

4 $${\text{Dry gluten, \% }} = 100 - \left( {\frac{{{\text{W}}1 - {\text{W}}2}}{{\text{m}}} \times 100} \right)$$

where: W1 = mass of the wet gluten and of the dish before drying (g), W2 = mass of the dry gluten and of the dish after drying (g), m = mass of the wet gluten weighed for drying (g).

### Statistical analysis

The values determined in the laboratory measurements were analyzed using different statistical methods. The results of the three measurements were averaged and the reliability of the mean was determined using the 95% confidence interval of Eq. (5)^20^.

5 $${\overline{\text{x}}} - {\text{t*}}\frac{{\text{s}}}{{\sqrt {\text{n}} }} \le {\upmu } \le {\overline{\text{x}}} + {\text{t*}}\frac{{\text{s}}}{{\sqrt {\text{n}} }}$$

where: ${\overline{\text{x}}}$ = represent the average, t = Student’s t, s = standard deviation, n = sample size.

Description statistics were applied to the different wheat flours by calculating the minimum, 25th percentile, median, mean, 75th percentile and maximum values for the samples. Furthermore, using the Microsoft Excel spreadsheet, we performed a box-plot analysis for the measured parameters^21^.

Hierarchical cluster analysis (HCA) was performed using the IBM SPSS statistical software and the clusters were plotted in the form of dendrograms. To construct the dendrogram, the following parameters were used: centroid cluster, squared Euclidean distance and standardization was applied to a value between − 1 and 1^22^. Spearman correlations were calculated for the tested wheat flour, using the R 64.3.6.3 software package.

## Results

### Descriptive statistics

Based on the obtained data (Table 2), the moisture content of the tested flours varied between 9.76 and 11.15%. The lowest ash content was found in case of Gobé 000 white flour (0.36%), while the highest ash content was found in Nagyi titka black flour (2.02%). Regarding the moisture content, all tested wheat flours had a moisture content below the permissible level (14.5%), which is an important parameter for long term storage of the product. The wet gluten content varied between 27.9 and 40.65%. The dry gluten concentration was calculated from wet gluten. Based on the dry gluten content, the average variation of the tested flour types was in the following order: 480 wheat flour (13.19%) > 550 wheat flour (12.89%) > 650 wheat flour (11.87%).

**Table 2:** Descriptive statistics of different types of flour.

U.M.		N	Moisture	Ash	Wet gluten	Gluten spread	Gluten moisture	Dry gluten
			%	% d. m	%	mm	%	%
Wheat Flour 480	min	11	9.76	0.36	27.90	0.00	49.59	11.24
	$\overline{{\text{x}} }$		10.44	0.52	31.79	1.33	58.75	13.19
	CI95% −		10.21	0.46	29.89	0.89	56.95	12.42
	CI95% +		10.68	0.55	33.68	1.78	60.55	13.97
	max		11.15	0.63	39.32	2.87	61.91	15.81
Wheat Flour 550	min	9	9.87	0.50	24.76	0.25	57.94	9.76
	$\overline{{\text{x}} }$		10.24	0.57	31.90	1.92	59.56	12.89
	CI95% −		10.12	0.55	28.57	1.08	59.02	11.59
	CI95% +		10.36	0.60	35.23	2.75	60.10	14.20
	max		10.55	0.63	40.65	4.67	60.71	15.97
Wheat Flour 650	min	8	9.65	0.53	22.34	0.00	55.57	9.19
	$\overline{{\text{x}} }$		10.10	0.63	30.02	0.77	60.39	11.87
	CI95% −		9.93	0.59	25.80	0.09	58.34	10.15
	CI95% +		10.28	0.67	34.24	1.45	62.45	13.59
	max		10.45	0.70	39.47	2.67	66.49	15.45
Black flour	min	11	8.3	0.6	25.2	0.0	56.4	10.33
	$\overline{{\text{x}} }$		9.59	1.37	*	*	*	*
	CI95% −		9.29	1.14	*	*	*	*
	CI95% +		9.89	1.60	*	*	*	*
	max		10.30	2.02	*	*	*	*

Where * the number of samples (4) in case of gluten analysis was not statistically sufficient.

### Box-plot analysis

**Figure 1:**
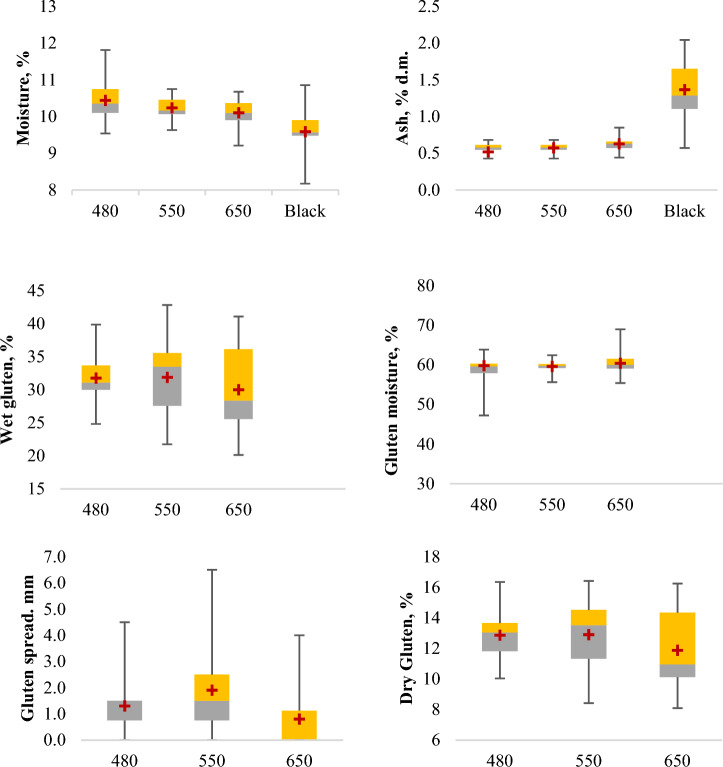
Box-plot analysis of the flour parameters. The lower (grey) and upper (yellow) limits represent the second and third quartiles, means are represented by red crosses, and the ends of the whiskers represent the minimum and the maximum values. The figures were prepared using the Microsoft Excel program.

In order to analyze data distribution box-plot analysis was carried out, where the minimum and maximum values represent the range and the width of the quartiles (Q2-grey and Q3-yellow) represent the occurrence of the patterns. In terms of moisture content, the highest concentration was found in white flour (480 White Flour > 550 White Flour > 650 White Flour), on the opposite side, the lowest moisture concentration was found in whole meal flour. Approximately the same high levels of moisture and dry matter concentration were observed for 550 and 480 wheat flour. Wheat flour 650 had a higher moisture content of gluten, resulting in a lower dry gluten content (Fig. 1).

### Classification of different types flour based on their wet gluten content

**Figure 2:**
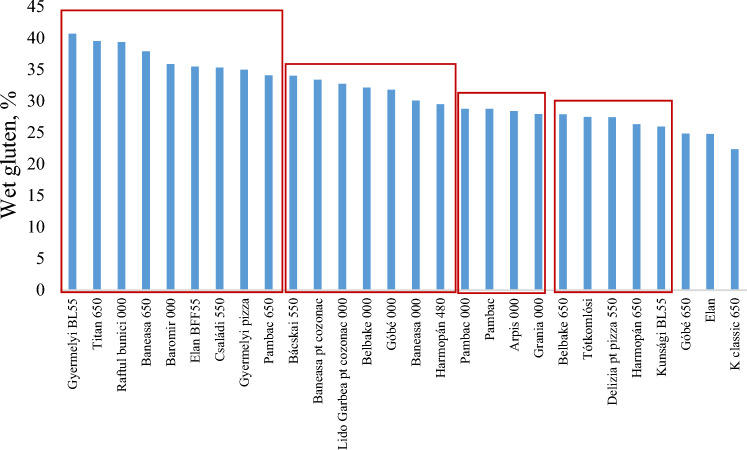
Classification of wheat flours based on the wet gluten content.

The results of the measurements show that 32.1% (9 pc) of the tested flours were classified as improving flours, hence the wet gluten content exceeded 34% for the following wheat flour types: Gyermelyi BL55, Titan 650, Raftul bunicii 000, Baromir 000, Elan BF550, Családi 550, Gyermelyi pizza, Pambac 650 (Fig. 2).

The second category includes almost a quarter of the flours, which are characterized by a wet gluten content higher than 30%. Flours with a high wet gluten content (> 34%) are suitable for improving the properties of lower quality flours.

### Classification of different types of flour based on the wet gluten spread.

**Figure 3:**
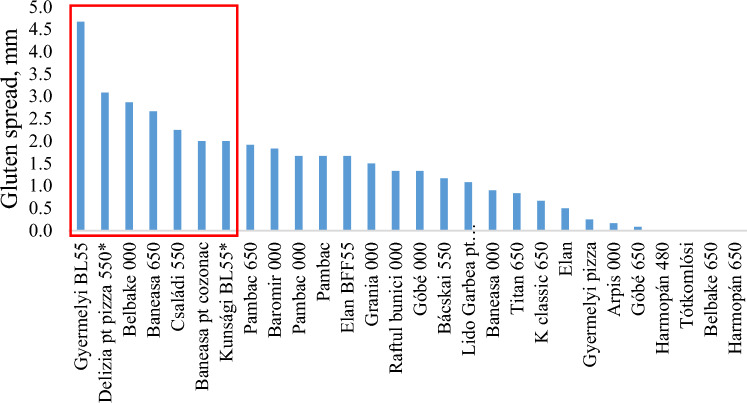
Classification of whet flours based on the gluten spread capacity.

Large differences were observed between the tested wheat flours, based on the gluten spread. The average gluten spread area of 7 flour brands (Gyermelyi BL55, Deliza pizza 550, Belbake 000, Băneasa 650, Családi 550 and Băneasa (bread), Kunsági BL55) can be considered as adequate, the area was between 2 and 5 mm. Taking into account the wet gluten content of these flours, it was also observed that the wet gluten content of Deliza for pizza 550 and Kunsági BL550 did not reach the threshold of good quality flours. In the other cases tested, the gluten spread was less than 2 mm (Fig. 3).

### Spearman correlation

**Figure 4:**
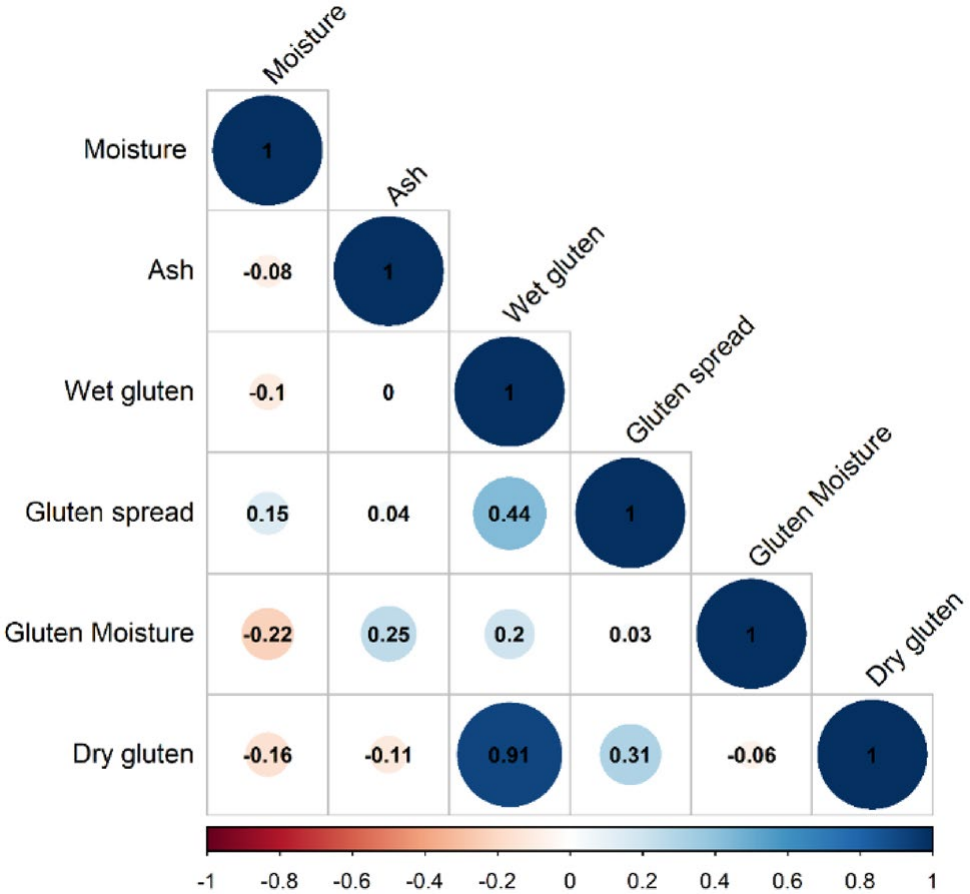
Spearman correlation of the studied flours parameters.

Based on the sample size (N = 31, P < 0.1), the significance level was set at r = 0.24. Given this significance level, a positive correlation was found between wet gluten and gluten spread (r = 0.44). Furthermore, a positive correlation was found between the dry gluten and gluten spread (r = 0.31). A significant negative correlation was found between the flour moisture and gluten moisture (r = − 0.22) (Fig. 4).

### Hierarchical cluster analysis

**Figure 5:**
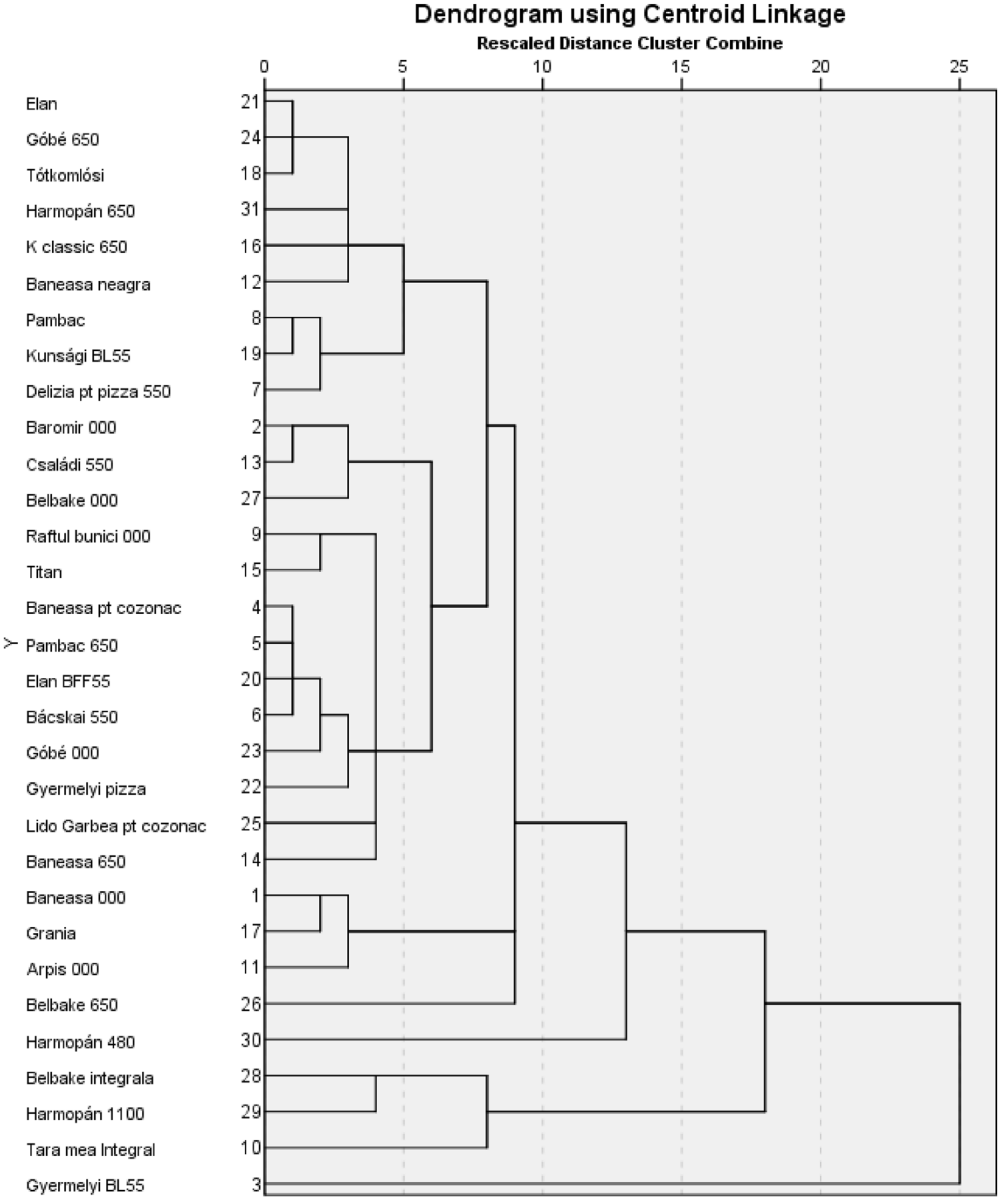
Hierarchical cluster analysis of the tested wheat flour.

According to the cluster analysis, the wheat flours are closer to each other in the cluster analysis in case of higher similarity, and further away in case of lower similarity. The hierarchical cluster analysis was carried out on the basis of the 31 studied wheat flours parameters. In the dendrogram (Fig. 5), it can be seen that all wheat flour, except Gyermely BL55, are connected in the first major cluster in different order. Sub-cluster 1.1 covers 87% of the analyzed samples. White flour (000, 550, 650) is located in this cluster 1. Sub-cluster 1.2 includes wheat flours with high ash content (Belbake, Harmopan, Țara mea).

## Discussion

In case of all studied flour samples, the moister level was below the maximum permission value (14.5%), which is as key parameter for the long term storage^23^. Based to the Codex Alimentarius Standards the maximum permissible level of moisture is fixed at 15.5%^19^. Flours with higher moisture levels are more susceptible to contamination, which can result in unpleasant odors and flavors, as well as mycotoxins infections^24^. The mineral content of the flours is given by the ash content, which is mainly influenced by two factors: cereal types and the milling process. The ash content of wheat varied between 1.5 and 2%, meanwhile the endosperm ash content was only 0.35%. Given that the endosperm makes up 80% of the wheat and the non-endosperm parts (pericarp, aleurone, and germ) have a high ash content, the ash content is strongly related to that of the non-endosperm part of the wheat. It is obvious that the non-endosperm part of the wheat has a negative influence on baking quality^25,26^.

Over the last century the ash content of flours has increased, from 0.45 to 0.55%. Our results are is accordance with those reported by Draghici et al.^27^, where they found that wheat flours moisture varied between 10.11 and 10.44%, and the gluten content also varied between 14.7 and 26.75% (Supplementary Figures 1-6).

The wet gluten content is an important qualityindicator of the flour, since there is a linear relation between gluten concentration and gluten forming proteins concentration, namely glutenins and gliadins. Furthermore, gluten is a key and essential parameter for bread making because influences the dough mixing and baking properties. Flours with high wet gluten content (> 34%) can be used to improve the properties of lower quality flours^28^.

## Conclusions

In the present study, the moisture, ash and gluten content (wet, dry, spread) of 39 wheat flours from the Romanian market were analyzed. Differences were found between the studied flour categories. White wheat flour was characterized by higher moisture, wet gluten and lower ash content. Furthermore, whole wheat flour had lower moisture and higher ash content.

The results of the laboratory determinations showed that the moisture content of the tested wheat flours was within permissible levels for extended storage, aligning with established standards, the moisture level varied between 9.5 and 11.8%. The ash content varied from 0.43 to 2.04 g/100 g. The average wet gluten content of the flours tested varied between 30 and 32%, and the average dry gluten content of the flours was 12.8%. This study serves as a valuable resource for both consumers and industry as it provides insights into the quality and variability of wheat flours, which can aid in informed decision-making and potentially facilitates improvements in product quality and consistency.

Further studies are needed to determine the carbohydrate, protein, dietary fiber and mineral composition of wheat flour in order to estimate the potential health effects attributed to the different cereal varieties.

## Recommendation

The protein to starch ratio is an indicator of the quality of wheat grain. The higher the protein ratio, the better the quality of the wheat. Additionally, the higher fiber content of the flour helps in maintaining and restoring normal intestinal function.

### Supplementary Information

Supplementary Figures.

## Data Availability

The datasets used and analyzed during the current study are available from the corresponding author on reasonable request.

## Abbreviations

Ash crude %: Crude ash content
Ash %: Ash content
CI 95%: Confidence interval 95%
CSO: Central Statistical Office
FAO: Food and Agriculture Organization of the United Nations
HCA: Hierarchical cluster analysis
Hum %: The samples humidity %
m: Mass of the sample weighed for drying (g)
m1: Mass of the crucible and sample after incineration (g)
m2: Mass of the crucible (g)
max: Maximum value
min: Minimum value
mo: Mass of the sample weighed for the test (g)
n, N: Sample size
Q1: First quartile
Q2: Second quartile
Q3: Third quartile
Q4: Fourth quartile
r: Correlation coefficient
s: Standard deviation
t: Student’s t
$${\overline{\text{x}}}$$: Average
W1: Mass of the sample and of the dish before drying (g)
W2: Mass of the sample and of the dish after drying (g)
