# Supplementary material for: Physical–chemical analysis of different types of flours available in the Romanian market
Source: Sci Rep. 2024 Jan 9;14:881. doi: 10.1038/s41598-023-49535-x (PMC10776669; doi:10.1038/s41598-023-49535-x)
Supplement: Supplementary file 1 — Supplementary Figures. [file 41598_2023_49535_MOESM1_ESM.docx]

**Supplementary material**

**Fig S1**. The studied wheat flours moisture content

**Fig. S2**. The studied wheat flours ash content

**Fig. S3**. The studied wheat flours gluten moisture

**Fig. S4**. The studied wheat flours wet gluten content

**Fig. S5**. The studied wheat flours gluten spread

**Fig. S6**. The studied wheat flours dry gluten content
